# Leaving no woman or girl behind? Inclusion and participation in digital maternal health programs in sub-Saharan Africa

**DOI:** 10.1186/s12978-022-01358-1

**Published:** 2022-02-28

**Authors:** Ogochukwu Udenigwe, Sanni Yaya

**Affiliations:** 1 School of International Development and Global Studies, Faculty of Social Sciences, University of Ottawa, Ottawa, Canada; 2 The George Institute for Global Health, Imperial College London, London, UK

**Keywords:** Digital health, Maternal health, Health inequity, Gender, Sub-Saharan Africa

## Abstract

Across sub-Saharan Africa where access to adequate maternal healthcare is fraught with myriad challenges, especially for hard-to-reach populations, digital health technologies offer opportunities to improve maternal health outcomes. Digital health can circumvent inefficiencies in the traditional healthcare system and address challenges such as limited access to in-person medical consultations, and poor access to skilled birth attendants and health promotion activities. These benefits notwithstanding, digital health can be exclusionary. Too often, digital maternal health programs are not designed with a focus on equity in distribution nor are they designed from a gender equity standpoint. In this paper, we illustrate exclusionary practices of digital health programs through an extensive literature review of digital maternal health programs across sub-Saharan Africa. Taking an intersectional approach, we discuss how women are most vulnerable and excluded at the intersection of gender, literacy, and disability. Tackling exclusionary practices in digital health is crucial to ensure that no woman or girl is left behind.

## Introduction

At the heart of the ambitious sustainable development goals (SDGs) is the commitment by member states to end poverty by “leaving no one behind” and to “endeavour to reach the furthest behind first” [1]. The SDGs recognise that prioritizing maternal, neonatal and child health is key to eliminating poverty and disparities in low-and middle-income countries. This is of particular importance in Sub-Saharan Africa where a majority of women are without access to essential healthcare during pregnancy and childbirth [2]. Systemic and persistent gaps in access to maternal healthcare services were reported across socio-economic and geographic lines [2]. The three main contributors to unequal access to maternal healthcare among women are wealth, education level, and area of residence (urban/rural). These socio-economic barriers are often interlinked; women from poor families are more likely to be uneducated and live in rural areas. Furthermore, limited access to maternal healthcare services is higher in instances where provider-patient interactions are required, for example, in antenatal care or delivery attended by a skilled birth attendant.

Measures put in place to curb the spread of COVID-19 have been shown to exacerbate the gap in access to maternal healthcare, particularly among the most vulnerable. Across sub-Saharan Africa, various studies show disruptions in the delivery of maternal healthcare services due to the diversion of healthcare resources to COVID-19 care [3, 4]. The rural–urban divide in access to maternal healthcare services has grown wider and some women remain unable to access maternal healthcare services due to financial challenges brought about by job losses during the pandemic [5]. The UN advocates for gender-inclusive responses and recovery measures during the pandemic including the sustenance of sexual and reproductive health services [6]. In prioritizing gender-inclusive response strategies, the UN recommends moving services online.

Online platforms particularly in the health sector have become increasingly important. The use of digital technology remains a key element of countries’ resilience and recovery as their health systems respond to shocks and disruption caused by the COVID-19 pandemic [7]. The WHO noted that digital health “plays a unique and pivotal role in achieving universal health coverage. It extends the scope, transparency, and accessibility of health services and health information. It widens the population base capable of accessing the available health services and offers innovative and efficient gains in the provision of health care” [8].

### Digital health for maternal healthcare

Across sub-Saharan Africa where access to adequate maternal healthcare is fraught with myriad challenges especially for hard-to-reach populations, digital health technologies offer opportunities to improve maternal health outcomes [4]. Digital health (specifically mobile health) looks at the use of mobile devices for medical and public health practices. For example, mobile devices such as mobile phones, patient monitoring devices, personal digital assistants (PDA), and wireless devices can be applied to a broad spectrum of health activities including telephone helplines or text message-based health information [9]. Digital health can circumvent inefficiencies in the traditional healthcare system and address challenges such as limited access to in-person medical consultations, and poor access to skilled birth attendants and health promotion activities. These benefits notwithstanding, digital health can be exclusionary.

Too often, digital health programs are not designed with a focus on equity in distribution nor are they designed from a gender equity standpoint [10]. Digital health programs would often require that beneficiaries have access to mobile devices without noting the uneven distribution in ownership of mobile devices (henceforth referred to as “mobile ownership”). There is a significant variation in mobile ownership between men and women across sub-Saharan Africa, this gap persisted even with the increased reliance on digital technologies during the pandemic [11]. Women, who are often key beneficiaries of digital health programs for maternal healthcare, are overall less likely to own or have independent control of mobile technologies. Across sub-Saharan Africa, country-level gender gaps in mobile ownership range from 4% in Nigeria (89% men mobile owners vs 86% women mobile owners) to 27% in Mozambique (64% men mobile owners vs 47% women mobile owners) [11]. This gender gap is further exacerbated by socioeconomic characteristics such as illiteracy and poverty whose negative effects are greater for women [12]. In such instances, digital health programs may not only miss their intended population but exacerbate inequality in access to healthcare services.

These unfolding arguments have implications for operationalizing digital health programs for maternal healthcare in sub-Saharan Africa. In this paper, we illustrate exclusionary practices of digital health programs through an extensive literature review of digital maternal health programs across sub-Saharan Africa. Taking an intersectional approach, we discuss ways in which women are digitally excluded and how the most vulnerable are still underserved in the digital health arena.

Table 1 indicates the types of digital maternal health programs included in this paper. This paper focuses exclusively on programs directed towards pregnant or postpartum women as direct beneficiaries and end-users; and reports majorly on programs that involve interactions between women and healthcare providers.

**Table 1:** Types of digital maternal health programs

Program Type	Description
Appointment reminders	Reminder messages to women from healthcare providers to attend appointments using mobile devices. Messages can be sent through SMS-based text, video, or multimedia messages
E-vouchers (mobile money)	Electronic vouchers to finance maternal healthcare services using mobile devices; involves the use of mobile apps
Healthcare telephone helplines	Healthcare advice to women through live phone interactions with healthcare providers or pre-recorded messages; accessible through mobile phones or landlines
Health information	Administering health information through mobile devices. Can be conveyed through uni- or bi-directional SMS-based text, video, or multimedia messages
Health promotion	Health promotion campaigns through mobile devices. Can be conveyed through SMS-based text, video, or multimedia messages
Treatment adherence	Messages sent to women to achieve treatment adherence. Can be conveyed through SMS-based text, video, or multimedia messages
Surveillance	Surveillance of patients to ascertain pregnancy and/or birth outcomes, obtained through mobile telephone calls to women or bi-directional text messages between women and healthcare providers

### Intersectionality in digital maternal health programs

In explaining poor maternal health outcomes across sub-Saharan Africa, feminist critiques have problematized patriarchy [13, 14]. Patriarchy underlies gender roles that reduce women’s access to material resources compared to men, as well as complex systems of power organised across institutions that restrict women’s rights and freedoms. Similarly, gender hierarchies within men and women foster discrimination against gender-diverse and non-conforming individuals [13]. Gender is, therefore, an important stratification in understanding determinants of maternal health. Beyond gender as a crucial category, it is important to recognise that women are situated differently across racial, economic, social, and political contexts. Maternal health reform efforts such as digital health, portray women as a homogenous group without acknowledging their intersecting identities and positions [15]. In so doing, only a select few experiences and perspectives are privileged while others are neglected and put at high risk of adverse health outcomes. Viewing maternal access to health services through an intersectionality lens sheds light on these connections between privilege and oppression.

Intersectionality as a theory draws attention to the multiple layers of disadvantage at societal and individual levels that impact health and well-being. The basic tenet of this theory is that an individual’s experience cannot be explored and understood by isolated categories of their identities, instead, experiences are shaped by multiple identities that interconnect and interact [10, 16, 17]. These interactions are key in producing experiences of oppression or privilege. Some feminist scholars conceptualise intersectionality through the intimate connections between privilege and oppression to encompass the different positionalities of marginalised women. For instance, some women can be privileged by race but victimized by patriarchy [17, 18]. Other feminist scholars highlight the positions of multiply marginalised women. Intersectionality, as developed and discussed by black feminists, critiques the erroneous assumption of isolated categorical axes of oppression such as gender, class, or race and instead highlights the complexity and interconnectedness between diverse modes of domination [16, 17, 19]. It considers that identities and systems of power can vary across different contexts be it geographic or political. Intersectionality theory is deeply rooted in advancing social justice and advocates for strategies that address inequities and reduce or eliminate vulnerabilities [20].

An intersectional lens in digital health emphasises diverse modes of oppression including but not limited to sexism, class oppression, racism, ableism, and heterosexism, that underlie intersecting systems of power that compound and exacerbate inequities and ill health [13]. For example, the impact and benefit of a digital health intervention for pregnant women in a sub-Saharan African context could be anchored on geographic binaries (rural vs urban settings) [12, 21]. Compared to a pregnant woman in an urban setting, a pregnant woman in a rural area is less likely to benefit from a digital health program due to limited mobile phone access despite having more barriers to accessing skilled maternal healthcare services [21]. Even among women with digital technologies within the same geographic location, vulnerabilities such as low literacy or disabilities could diminish the impact of a digital health program [21]. Thus, intersecting factors such as socioeconomic status, literacy level, and disabilities are crucial to women’s access to and use of digital health technologies for maternal healthcare [10]. In the next section, this paper discusses how these systems of oppression are further used as grounds for exclusion in digital health.

### Exclusionary practices in digital maternal health programs

#### At the intersection of gender and mobile ownership

The literature suggests benefits to using digital technology for maternal healthcare tasks ranging from text messaging as a notification system, or as a form of health education, to tools for data collection on maternal health information [22, 23]. However, experiencing these benefits, even for those with the greatest healthcare needs, depends largely on access to mobile devices.

A recent review of mobile health initiatives across sub-Saharan Africa indicates that compared to women with access to mobile devices, women with no mobile access have limited health knowledge and are significantly less likely to receive skilled maternal care services including antenatal care, delivery care, and postpartum care [24]. This suggests that the digitally excluded women have a greater need of services offered by digital health technologies yet are being left behind. This consideration brings under scrutiny the elusive participation criteria in digital maternal health programs.

From a sub-Saharan Africa context, a major criterion for participation or inclusion in digital maternal health programs is mobile ownership and independent control of a mobile device (often phone) [25–28]. This criterion inevitably excludes women who may not own mobile phones but have access to shared devices. Another restrictive participation criterion is a focus on internet access. For instance, a digital maternal health intervention study in South Africa required participants to have access to a smartphone and internet even after noting that only half of the general population has access to smartphones and reliable internet [29]. There was no indication of gender-specific considerations in mobile ownership or access to the internet. This criterion is often further complicated by the requirement to be connected to specific internet providers [30–32]. These criteria dictate who reaps the intended benefits of digital health programs and who is left behind.

Mobile devices, though a necessity, are not ubiquitous [33]. However, this point is seemingly neglected in digital health practices and reflects, to a large extent, the social structures and systems of power that govern their design and use. The sociology of digital technologies is largely informed by men, and this is reflected in the wider socio-cultural and economic context within which they are accessed and used [10, 12, 33]. Men’s dominance in digital technology is woven into its various facets including infrastructure, decision-making and leadership, app design, and algorithms [10, 12, 33]. Therefore, gender-specific constraints to women’s active engagement with digital technology are largely ignored. In fact, a widely pervasive assumption is that digital technology is gender-neutral, therefore men and women use and benefit from it equally [12, 33]. Arguably, digital maternal health programs demanding mobile ownership as a criterion for inclusion and participation are assuming gender neutrality in access to and use of mobile devices. Not only is this approach engendering gender inequality, but it also risks leaving behind the most vulnerable women.

In contrast to restrictive participation and inclusion criteria, some digital maternal health programs reported on inclusive strategies to enhance women’s engagement in digital health, particularly for women without mobile ownership. For instance, digital health programs in South Africa and Kenya have taken a pragmatic approach and included as their participation criteria women who have access to a mobile phone through a family member or friend [34–38]. While there is some evidence to show that mobile phone sharing is a common practice in some sub-Saharan African settings, critics view this strategy as a limitation [39]. A major critique of this approach hinges on privacy concerns [23, 40]. For example, pregnant women living with HIV who were also recipients of health information via text messages feared inadvertently disclosing their status if their text messages were read by a third party [41, 42]. Understandably, restrictive criteria are necessitated by privacy concerns particularly when it involves sensitive health topics. However, some digital health programs are circumventing privacy concerns. One digital health program reportedly pivoted from sending sensitive text messages to voice calls, this allowed women the privacy of discussing their health concerns with their healthcare providers rather than having them written down [43]. To prevent privacy breaches, other programs have considered password-protected health apps or auto-lock functions that activate immediately after use for women using shared mobile devices [40].

Furthermore, some digital health programs, borne out of the cognisance of the low rates of mobile ownership among women, offered alternatives to participating in digital health programs. In Nigeria, a digital maternal health program offered group mobile phone-based programs to pre-existing community groups instead of programs based on individual mobile ownership. The study was able to reach the poorest and least educated women who were also less likely to own mobile phones or engage in optimal health practices [44]. This approach was deemed feasible and acceptable by participants. In another instance, women without access to mobile devices were offered in-home services whereby community health workers brought mobile devices to women’s doorsteps to disseminate health information [45].

#### At the intersection of gender and literacy

Beyond the constraint of gender inequality in digital technology ownership, issues of access to digital health technologies are exacerbated by low levels of literacy. The basic idea of literacy refers to having reading, writing, and numeracy skills [46]. Much of the requirement for participation and inclusion in digital health programs require basic literacy, although this could also extend to other domains including digital literacy. Digital literacy refers to the basic skills and competencies required to use digital technologies to access, manage and use information, produce new knowledge, and communicate with others [46]. Around the globe, individuals with low levels of literacy are concentrated in South East Asia and sub-Saharan Africa; women make up about two-thirds of this group [46]. As of 2020, only 59% of adult women in sub-Sahara Africa were literate [47]. At country levels, these rates range from 95% adult female literacy in South Africa to 14% in Chad [47]. Studies have highlighted the unique health circumstances of low-literate women by establishing an association between low literacy rates and excessive maternal healthcare costs, higher maternal mortality, and negative healthcare outcomes [48, 49]. This segment of women, who are undoubtedly most in need of digital health services, are less likely to enroll in them [26, 50].

Yet, an overwhelming number of digital maternal health programs in sub-Saharan Africa list literacy as an inclusion criterion [28–30, 32, 51–53] with no purposefully designed solutions to help women with limited literacy skills navigate the digital health space. In instances where literacy was not an explicit exclusion criterion, low literacy was associated with low engagement with the digital health program [50]. This suggests that even when literacy is acknowledged as a barrier to accessing digital health programs, certain challenges linger. For instance, health-related information may not be conveyed in simplified terms, or digital health programs may be too sophisticated in their design and require advanced digital literacy skills [46].

Another conspicuous challenge was the exclusion of participants based on language competencies. Some digital health programs included as a participation criterion, the ability of women to read and speak in English. This was observed even in settings where English was not considered the official language or language of the majority [32, 51]. For instance, a study noted that while English was a preferred choice of the majority in the population, the study did not reach women at a greater disadvantage due to language barriers [32]. A general assertion in digital health is to provide services in a country’s national language or in a common language that serves the majority [54]. This is problematic in a continent such as Africa that is reputed for its multiple languages so much so that a national language or one considered to be “commonly spoken” by the majority is in fact truly common to only about 20% of the population [55].

These criteria for participating in digital maternal health programs suggest a failure to acknowledge the disparities across the target population and a flawed assumption that the benefits of digital health interventions are universal [12]. This poses a challenge for the most underserved and excluded women. Viewing digital maternal health through an intersectional lens would extend beyond serving the majority and instead acknowledge the unique challenges faced by women at the intersections of overlapping systems of power such as women with low literacy levels who do not speak their country’s dominant language.

Some digital health programs illustrated efforts to enhance the accessibility and inclusivity of their programs. For instance, literacy was not a participation criterion for some programs provided women had someone who could read messages or operate mobile devices on their behalf [35, 56–59]. Other digital health programs acknowledged low levels of literacy in their target population and augmented text-based informational programs with videos and pictorial messages [58]. This strategy also worked to overcome language barriers.

#### At the intersection of gender and disabilities

While inclusion and participation criteria in digital maternal health programs are subjects of intense inquiry in this paper, accessibility considerations for women with disabilities are an equally important subject of scrutiny. In a sub-Saharan Africa context, women with disabilities continue to face barriers to maternal health services. They are largely ignored in reproductive and maternal health research studies and programs and can be argued to be the ones most “left behind” in digital health [46, 60]. Compared to non-disabled women, women with disabilities face an increased risk of multiple forms of violence, are more likely to have lower educational attainment, poorer health outcomes, limited economic opportunities, and have higher rates of poverty [61]. When viewed from an intersectional lens, women with disabilities encounter all of the above and still face physical, structural, and attitudinal barriers that infringe on their reproductive rights [62]. They have poor access to maternal healthcare services and are at risk of having poor maternal health outcomes. Digital health solutions can help women with disabilities overcome these intersectional challenges. However, they are more likely than non-disabled women to be digitally excluded and unable to access digital health services for maternal care. The digital divide experienced by women with disabilities is severely under-researched and there remains a paucity of data on their unique challenges particularly in a sub-Saharan African context.

Disability is, broadly defined by the World Health Organization as physical, cognitive, speech, sensory, mental, emotional, and developmental impairments that place limitations and restrictions on an individual’s social participation and environmental access [60, 61, 63]. It is important to note that in the context of maternal health, health disparities observed among people with disability is a result of being denied maternal health services. As an example, studies in sub-Saharan Africa have cited the pervasive assumption of women with disabilities as asexual or not likely to have children thereby limiting the number of programs tailored to their health needs [60]. They are also more likely to be met with hostile and insensitive attitudes from healthcare providers [64].

There is limited evidence in sub-Saharan Africa’s digital maternal health literature to show inclusive considerations for women with disabilities. There were no mentions of considerations made for women with hearing or vision impairments including the deaf and deafblind. While considerations were made for women with limited literacy in terms of pivoting to multimedia messages instead of text-based messages, there were no mentions of accessible visual presentations adjusted for size or the use of colours for women with visual and sensory impairments. Furthermore, while some women may have access to mobile devices, they may face dexterity impairments that limit their use of mobile devices and associated apps. There was no mention of this as a consideration in digital maternal health programs.

### Moving towards inclusivity: recommendations and conclusion

These observations highlight the growing need to ensure that the benefits of maternal digital health strategies are equitably distributed. It has been shown that the use of digital health can lead to better health outcomes, better patient experiences, and better value of care for the most vulnerable population and in hardest to reach areas [54, 63]. These would not be realised without widening the population capable of accessing digital health services. Addressing the aforementioned barriers that continue to exclude women from the benefits of digital health- ownership, literacy, and disabilities—requires action from multiple stakeholders. This section of the paper includes recommendations intended for researchers, digital health implementers, policymakers, and advocates to facilitate equitable access to digital health services for maternal healthcare and to ensure that no woman is left behind.

#### Enhancing mobile ownership

The persistent gender digital divide is in part driven by affordability. Across sub-Saharan Africa, affordability was a major barrier to women owning mobile devices [11]. This is because women are more likely than men to have lower incomes and lower financial autonomy which limits their ability to purchase mobile digital devices. Improving the affordability of mobile devices will go a long way to bridging the gender divide in mobile ownership. A viable step towards increasing access to digital maternal health services for the most underserved women is to provide free or subsidised mobile phones to women in target populations who are less likely to own mobile devices [65]. Studies have supported the hypothesis that giving women free or subsidized digital devices improves their use of health care services and in turn, reduces maternal mortality [66]. Other stakeholders such as internet companies can ensure affordable access to digital health services by creating low-cost connectivity options without compromising on quality such as data light options for health apps [11]. Policymakers must implement and support initiatives in their communities that help make mobile devices affordable for women.

To address the gender digital divide, efforts must go beyond ensuring women’s access to digital technologies. Women should not be confined to spectators and consumers of digital health technologies. Therefore, alongside increasing women’s access as users, women must be designers and leaders in the digital health spaces. Communities must challenge social norms that act as barriers to women using and accessing digital technology. This tilts the power differential in a patriarchal society and increases the likelihood that women’s needs and priorities are reflected in digital health services for maternal healthcare.

#### Digital inclusion of women with low literacy

Furthermore, an intersectional lens to digital health programs is needed to amplify the needs of often neglected and excluded women. It would be unwise to assume that owning mobile devices alone could redress inequalities that persist among women groups such as women with limited literacy skills. Implementers should always be cognizant of the multiple social disadvantages that are experienced across different axes, many of which can be experienced simultaneously. Furthermore, strategies to reduce privacy breaches such as password-protected health apps or auto-lock functions can enhance women’s participation and inclusion in digital health services in cases where women are confined to shared ownership of mobile devices.

A patient-centred approach in the context of digital health is imperative to understanding the barriers and facilitators to women’s use of and access to digital health services. A patient-centred approach “is rooted in active collaboration between patients and researchers” [67]. This often entails much research and interaction with end-users to receive critical, honest, and regular feedback. However, researchers should be cautious of the perceived power differential between them and the end-users. They might feel intimated by the researcher’s perceived social status and are prone to response bias. Furthermore, digital health developers are usually not members of communities where their programs are used and may fail to fully comprehend the cultural complexities involved with digital health programs’ access and use. Partnering with intermediaries who are embedded in the community such as women’s groups, is critical to enhancing social and cultural acceptance of digital services as well as to enhancing a patient-centred approach to digital health. Digital health implementers ought to assess the literacy and digital literacy skills of their target population and make plans to support participants according to their skill levels. The use of multimedia promotes the use of digital health platforms by women with different proficiencies and competency levels. For instance, digital maternal health programs can present text messages alongside graphics or videos thereby removing the requirement for end-users to have the ability to read or write in order to participate.

#### Digital inclusion for women with disabilities

Given the paucity of data on the technological needs of sub-Sahara African women with disabilities in a maternal healthcare context, there needs to be more research and data generated in this regard. Crucial to research and data generation is a deep understanding of the intersectionality of gender, literacy, and disabilities. The availability of data will inform action and policy directed at addressing the needs of women with disabilities in general and in the context of sexual and reproductive health, specifically. It is also important to raise awareness of digital products and services that address the needs of women with disabilities [68]. Beyond awareness, these products and services need to be accessible and affordable. Processes of designing digital health programs will need to align with lived experiences of women with disabilities, this would mean involving them early in the design and implementation process and being mindful of issues of trust and critical feedback.

African countries are seizing the opportunities offered by digital technologies. At least 42 of Africa’s 54 countries currently have a national digital health policy [69]. While this is a positive step, policies must be implemented from a gender and equity perspective to ensure the benefit of digital health for all.

## Abbreviations

SDGs: Sustainable Development Goals
UN: United Nations
WHO: World Health Organisation
